# Early U.S. Military Immunization Against Tetanus and Diphtheria: Historical Context and Current Importance

**Published:** 2025-08-20

**Authors:** G. Dennis Shanks

**Affiliations:** Australian Defense Force Infectious Disease and Malaria Institute, Gallipoli Barracks, Enoggera, Queensland; University of Queensland School of Public Health, Brisbane

## Abstract

Prior to the Second World War, toxoid immunizations for both tetanus and diphtheria had been developed but were not widely used in adults. Starting in 1941, however, the U.S. Army began extensive immunization with tetanus toxoid. Tetanus decreased dramatically, with only 12 tetanus cases (1 case per million) developing during the war, mostly in imperfectly immunized soldiers. Diphtheria immunization was more complicated, as many adults in 1941 had some natural anti-toxic immunity to diphtheria. A decision to not immunize the U.S. military against diphtheria was made due to low prevalence of the disease and high rates of adverse events. During the war, however, unexpectedly high rates of debilitating cutaneous diphtheria were seen in desert and jungle warfare, among prisoners of war, and amid epidemics of respiratory diphtheria in Europe. Those diphtheria cases resulted in the requirement for U.S. soldiers to be immunized starting in 1945. Adjusted toxoid doses post-war eventually arrived at an accepted dual toxoid regimen. Mass immunization remains the best prevention against diphtheria.

*It was rather a surprise to learn that diphtheria was an important and widely prevalent tropical disease* . ^1^ 1946

Vaccination of the U.S. Army began during the War of 1812, with Jennerian smallpox immunization substituting for variolation. A century elapsed before a second vaccine, for typhoid immunization of soldiers, began in 1912, following the Spanish American War. ^2^

The third standard U.S. Army immunization was tetanus toxoid, a chemically inactivated toxin that is immunogenic but not toxic. Vaccination of U.S. Army personnel with tetanus toxoid began in 1941, in the months preceding U.S. involvement in World War II. This immunization program resulted from successful tetanus toxoid reports from the British and French armies, with approval requiring nearly a year of effort by the U.S. Army Surgeon General's Office. ^3^

Due to the low prevalence of respiratory diphtheria at the beginning of World War II, immunizing the U.S. Army with diphtheria toxoid, although medically possible, was determined to be militarily impractical. ^3 , 4^ The decision not to immunize the U.S. Army of the Second World War against diphtheria toxoid was a practical, short-term measure with negative consequences. Surprisingly large numbers of soldiers in both the North African desert and South Pacific jungles were incapacitated by chronic skin ulcers caused by *Corynebacterium diphtheriae* , and over 120 U.S. Army deaths were directly attributed to respiratory diphtheria. Ultimately, immunization against diphtheria was begun in 1945, due to large European diphtheria epidemics. ^5 , 6^

## Tetanus

Tetanus was a major military problem in the trenches of the First World War, requiring literally millions of doses of equine anti-toxin for the wounded. ^9^ The development of tetanus toxoid immunization in the 1920s was a technological break-through, allowing individuals to produce their own antisera. Wound prophylaxis against tetanus became toxoid boosting and not equine antisera. Purification of the immunogen to eliminate peptone products used in bacterial culture eliminated some allergic reactions ^3^ ; this was in an era before randomized clinical trials, with evidence of efficacy largely based on comparison groups.

**FIGURE 1.**
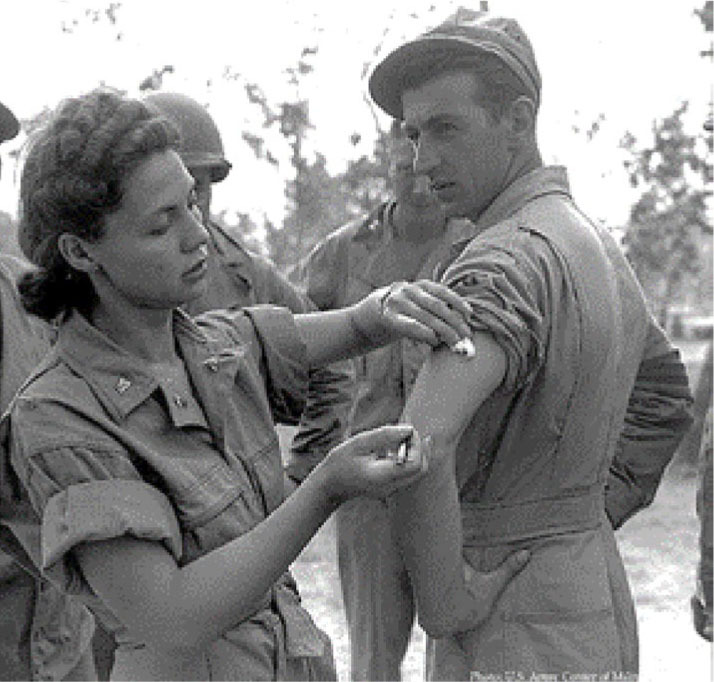
Army Nurse Immunizes U.S. Soldier, Probably Against Tetanus, Queensland, Australia, 1942

With the onset of another global war, the U.S. Army authorized tetanus toxoid in 1941 for overseas service, and then for all troops, after a year's discussion of the proposed policy change. ^2 , 3^ Despite logistical challenges, vaccination of millions of soldiers with tetanus toxoid was largely accomplished with few problems or adverse events **(Figure 1)** . The U.S. Army had 70 tetanus cases per million soldiers in the First World War compared to only 12 cases, most not fully immunized, or 1 per million soldiers, during the Second World War. ^3^

The U.S. military problem of tetanus during World War II was largely solved by consistent toxoid immunization of the entire population. ^2 , 4^ As neither the German nor Japanese Armies routinely immunized against tetanus, most clinical tetanus during the Second World War occurred in prisoners of war. During the fighting around Manila in 1945, 473 tetanus cases, with 389 deaths, were described in Japanese Army prisoners, but none occurred in wounded U.S. soldiers. ^3^ The Japanese Army belatedly attempted to develop its own tetanus vaccine in Jakarta, but inadequate inactivation of the toxin resulted in 900 iatrogenic deaths in Indonesians who served as unwilling product recipients. ^10^

## Diphtheria

Diphtheria toxoid had been developed in the 1920s and successfully prevented pediatric disease, as well as decreasing need for equine anti-diphtheria toxin. Respiratory circulation of toxigenic *C. diphtheriae* meant that in 1940 many U.S. adults had some immunity, as measured by the intradermal Schick test. Negative Schick results denoted an ability to neutralize a small intradermal dose of diphtheria toxin; positive tests indicated skin reactions with no antibody neutralization. ^11^

On the basis of Schick testing of 3,000 soldiers, in 1940 the U.S. Army calculated that a majority (55%) of soldiers already had some pre-existing diphtheria immunity. ^3^ Mass screening of soldiers with Schick tests was decided to be medically possible but militarily impractical. ^4^

Diphtheria vaccination was much more complex than tetanus vaccination, despite similarities in toxoid technology. Schick-negative individuals (i.e., with diphtheria antibodies) often had adverse reactions when immunized with diphtheria toxin, contributing to the resistance to mass vaccination of soldiers. Adverse events in Schick-negative soldiers included swollen arms and lost duty days, with hospitalization of several who were immunized.

This weighing of risks versus benefits was reasonable, based on the information available at the time, but it assumed a static environment, which is not typical of infectious disease epidemiology during war. There were only 122 cases of respiratory diphtheria in the U.S. Army in 1942, but by 1945 cases had increased to 3,455, mostly in Europe. ^3^ Mortality in the U.S. Army also markedly increased, with 86 of 125 total diphtheria deaths in 1945 occurring overseas. ^3^

During the war, epidemics of cutaneous diphtheria in soldiers in austere environments, such as the deserts of North Africa and jungles of the Southern Pacific, were occurring. ^1 , 16^ Desert or veldt sore was a diagnosis well-known from the First World War and returned to be problem during the Second World War, initially in the British Army in Palestine and then in the U.S. Army in the Pacific. ^16 - 18^ Chronic, debilitating ulcers resulted from toxigenic skin infections that healed very slowly. Some soldiers were removed from duty for months of rehabilitation due to chronic foot ulcers and had to be evacuated to specialist tropical disease hospitals in the U.S. **(Figure 3)** .

**FIGURE 2.**
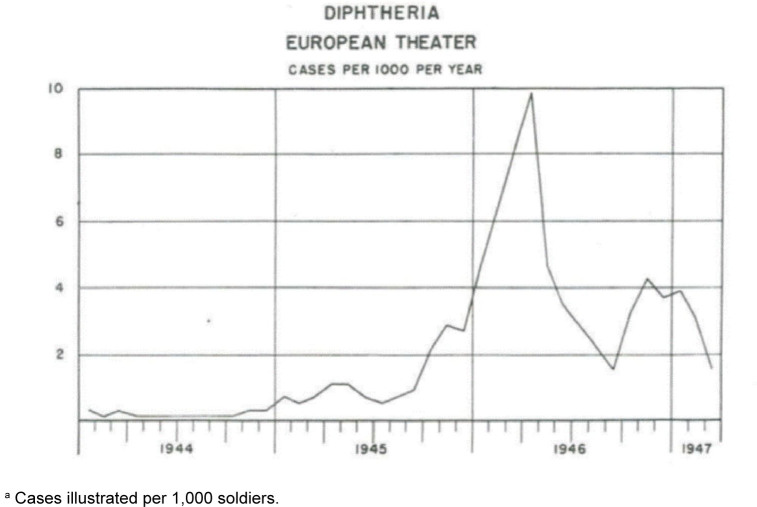
Incidence of Respiratory Diphtheria in the U.S. Army, Europe, 1944–1947 ^a^

**FIGURE 3.**
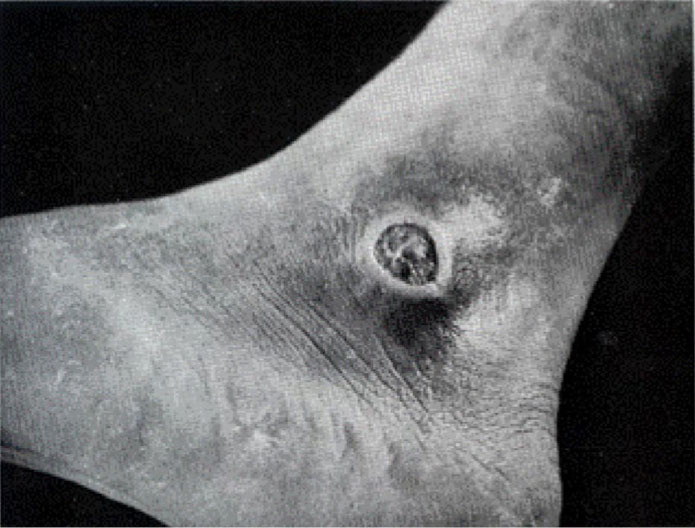
Chronic Skin Lesion Typical of *Corynebacterium diphtheriae* , U.S. Army Soldier Evacuated from Solomon Islands

Cutaneous diphtheria was probably the worst in Allied prisoner of war camps (PoW) in Asia, where debilitating tropical skin ulcers, compounded by starvation and other infections, often began a downward cycle to prisoner demise. ^19^ German PoW camps in the U.S. and U.K. also had problems with cutaneous diphtheria in unimmunized prisoners. ^20^

The massive, war-associated diphtheria epidemics during the Second World War led the U.S. Army to institute diphtheria toxoid vaccine in 1945 after Schick testing occupation soldiers, and then their families, in Germany. ^13^ Post-war diphtheria rates in the U.S. Army approached 10 per 1,000 per year in 1946, when diphtheria accounted for 15% of all medical deaths and 45% of all infectious disease deaths **(Figure 2)** . ^14^

Universal immunization against diphtheria in the U.S. military did not occur with mass Schick testing, but instead resulted from decreased immunogen in the standard combined tetanus-diphtheria toxoid vaccine (dT). The new formulation was accomplished by a series of studies among U.S. Navy recruits at Great Lakes Training Center. ^12 , 15^

Pharyngeal diphtheria, leading to systemic intoxication and potentially lethal myocarditis and neuritis, was effectively prevented by immunization and largely ceased to be a problem as universal dT immunizations became the standard both in children and adults after World War II. ^2^

## Commentary

Immunization with established toxoid vaccines eventually solved the military problem of exposure to toxigenic environmental bacteria. During the First World War, tetanus was a greatly feared disease that resulted from battlefield wounds. Nearly all medical officers had some experience with the often lethal disease. ^9^ The opportunity to dispense with reactogenic equine antisera and introduce tetanus toxoid immunization was embraced by Army leadership. Although general tetanus immunization of the U.S. Army began in the months before U.S. entry into the Second World War, requirements for diphtheria vaccination were more complicated, and were delayed due to adverse events and difficulty with mass Schick testing of soldiers during mobilization.

The delay against universal diphtheria immunization during World War II had 2 adverse consequences: 1) hundreds of frontline infantry soldiers in both Africa and Asia were incapacitated by chronic skin ulcers that healed poorly because of infection with toxigenic *C. diphtheriae* and 2) soldiers had to be immunized while deployed at the end of World War II once diphtheria became a major epidemic disease in Europe.

Few modern medical officers have any experience with what many now think to be extinct diseases, despite the perpetual presence of those pathogens in our environment. Tetanus is still a problem in unimmunized populations. ^21^ When public health systems failed to deliver universal toxoid immunization, diphtheria epidemics resulted, as seen in the former Soviet Union and Yemen. ^7 , 8^ When health systems collapsed in failed states such as Yemen and in conflict border areas of Pakistan, epidemic diphtheria resulted. ^8 , 22^

Fear of adverse events and practical issues in screening soldiers for pre-existing immunity against other diseases exists today, but failure to continue current toxoid vaccination policies could once again threaten U.S. soldiers with preventable illnesses from ubiquitous environmental bacterial toxins. Force protection measures, specifically immunization, must be maintained if the U.S. Army is not to rediscover the effects of diseases such as tetanus and diphtheria.
